# Systematic Review of Psychological and Behavioral Correlates of Recreational Running

**DOI:** 10.3389/fpsyg.2021.624783

**Published:** 2021-05-07

**Authors:** Hugo Vieira Pereira, Antnio Labisa Palmeira, Jorge Encantado, Marta Moreira Marques, Ins Santos, Eliana Veiga Carraa, Pedro J. Teixeira

**Affiliations:** ^1^Centro Interdisciplinar Para o Estudo da Performance Humana (CIPER), Faculdade de Motricidade Humana, University of Lisbon, Lisbon, Portugal; ^2^Centro de Investigao em Desporto, Educao Fsica, Exerccio e Sade (CIDEFES), Faculdade de Educao Fsica e Desporto, Universidade Lusfona, Lisbon, Portugal; ^3^Applied Psychology Research Center Capabilities and Inclusion (APPsyCI), ISPA - Instituto Universitrio, Lisbon, Portugal; ^4^ADAPT SFI Research Centre and Trinity Centre for Practice & Health Care Innovation, Trinity College Dublin, Dublin, Ireland; ^5^Laboratrio de Nutrio, Faculdade de Medicina, Universidade de Lisboa, Lisbon, Portugal

**Keywords:** physical activity, exercise, antecedents, psychological outcomes, behavior

## Abstract

**Introduction:** The aim of this review was to systematically synthesize the published literature describing the psychological and behavioral correlates of recreational running in adults, defined as running for leisure, with or without a competitive component.

**Methods:** Quantitative research published in peer-reviewed journals until January 2021 were included. Studies were identified through MEDLINE, PsycINFO, SPORTDiscus, and Web of Science and were included in this review if they (1) were aimed at recreational running, (2) included general adult samples (18 years or older, without a diagnosed medical condition or metabolic disorder), and (3) assessed psychological or behavioral correlates of recreational running.

**Results:** Fifty-six articles reporting 58 studies met the eligibility criteria and were included. There were 27 cross-sectional studies, 12 longitudinal studies, and 19 trials (8 non-controlled trials, 5 controlled trials, and 6 randomized controlled trials) (*n* = 37,501, 1877 years old, 43% women). Twenty-eight studies assessed antecedents of running behavior, and 25 studies used running behavior as treatment or predictor of a given effect or outcome. Four studies examined both predictors and outcomes of running. Thirty-one studies showed poor quality, while 20 had fair and 7 good quality. Motives were the most frequently studied antecedent of running behavior (*k* = 19), and results suggest that the highest-ranked or more prevalent motives were physical health, psychological motives, and personal achievement. Additionally, perceived control, attitude toward running, intention and subjective norms, self-efficacy, and social support may have also played a role in the adoption of recreational running. Moreover, improvements in mood (*k* = 10) and well-being (*k* = 10) were the most frequently reported positive outcomes of running. Reductions in depression, anxiety, and stress were also reported in included studies.

**Discussion:** To our knowledge, this is the first systematic review on this topic. The identification of behavioral and psychological correlates of recreational running across populations can contribute to inform and guide a public policy agenda, focused on helping people sustain regular physical activity, through a modality they have chosen and appear to enjoy.

**Systematic Review Registration:** https://www.crd.york.ac.uk/prospero/display_record.php?RecordID=68954, identifier: CRD42017068954.

## Introduction

Recreational running, defined as running for leisure, with or without a competitive component has increased exponentially (Scheerder et al., 2015). Although determined with different criteria, running prevalence in European countries varies between 31% in Denmark (considering running regularly in the last 12 months), 19% in Belgium (considering running as a leisure-time sports activity) and France, 18% in the Netherlands (considering participating at least once a year in a running activity), 15% in Finland, 13% in Germany, 6% in Spain, and 5% in the UK (considering running for at least four occasions in the previous 28 days, for at least 30 minutes, at moderate or vigorous intensity) (Scheerder et al., 2015). Portuguese adults indicated that running was the preferred leisure-time PA for 18% of men and for 8% of women (Teixeira et al., 2019), and the overall prevalence of recreational runners (considering at least two sessions and 60 min per week) was 10.6% (Pereira et al., 2021). Regarding non-European countries, there was a rate of participation in running and jogging activities of 16% in Australia (ASC, 2016) and 15% in the USA (Running-USA, 2017). Many factors contribute to the growth of recreational running, including the physical and psychological health benefits (Lavie et al., 2015; Nezlek et al., 2018), the low cost, and the fact that it can be performed in various contexts and requires little technical skills. The health benefits of running are vast, including prevention of obesity, hypertension, dyslipidemia, type 2 diabetes (Lavie et al., 2015), reduction of cardiovascular, and all-cause mortality (Lee et al., 2014) but also cancer mortality (Pedisic et al., 2019).

Recreational runners tend to run often, for more than 5 kilometers, and all year long (Bell and Stephenson, 2014), in many cases reporting many hours of training (Zach et al., 2017). This suggests the existence of potentially unique motivational and behavioral factors related with running and training for and completing a recreational race (Zach et al., 2012).

Most of the running-related literature focused on injuries (van der Worp et al., 2015), addictions (Hausenblas et al., 2017), or health-related outcomes (Pedisic et al., 2019), but understanding the interrelated psychological and behavioral attributes that explain *why* some individuals are regular (often avid) runners is also of importance to physical activity and public health research. Identifying running correlates, both antecedents and outcomes can contribute to more effective and tailored intervention approaches to promote the adoption and sustainability of recreational running.

One review has previously examined determinants for running, but with the purpose of developing a self-report questionnaire, and this was conducted using a systematic and comprehensive approach (Masters et al., 1993). Further, that work was published in 1993, and many primary studies (cross-sectional, longitudinal, and experimental) have been conducted since then (e.g., Tjelta et al., 2017; Malchrowicz-Moko et al., 2018).

The aim of this systematic review was therefore to identify and summarize the published literature describing psychological and behavioral correlates of recreational running in adults.

## Methods

This systematic review was conducted in agreement with the Preferred Reporting Items for Systematic Reviews and Meta-Analyses (PRISMA) statement (Liberati et al., 2009) and the protocol preregistered (PROSPERO: CRD42017068954).

### Eligibility Criteria

Studies examining psychological and behavioral correlates of recreational running in healthy adults (18 years or older), excluding preparation for any competitive sports, and without a diagnosed medical condition or metabolic disorder, were included. Studies conducted in samples of recreational runners only, and studies including both runners and non-runners' groups, were included. Observational and experimental design studies were included with no restrictions on the setting (e.g., community). Studies had to report a quantitative estimate for the correlate(s). Study protocols, reviews, and commentaries were excluded. This review was limited to articles written in English and published in peer-reviewed journals.

### Search Strategy and Study Selection

Electronic databases (MEDLINE, PsycINFO, SPORTDiscus, and Web of Science) were searched for relevant articles published between the review from Masters et al. (1993), finished in December of 1991 and January 2021, by combining keywords related with behavioral and psychological correlates of running behavior. Searches included a combination of four sets of terms: (i) terms concerning the population of interest (e.g., recreational runners), (ii) terms concerning the running behavior (e.g., jogging, marathon); (iii) terms related to antecedents of the behavior (e.g., self-efficacy, motives); (iv) and terms representing outcomes of running (e.g., mood, flow) (see Table 1 for the full search strategy). In addition, reference lists from previous reviews and retrieved papers were hand-searched to find additional studies.

**Table 1:** Full search strategy.

**Population**	**Behavior**	**Correlates**
NOT (injuries OR disease)	(recreational running OR recreational runners OR jogging OR jog OR marathon)	(motivation OR reasons OR intention OR regulations OR motives OR goals OR gains OR vitality OR happiness OR wellbeing OR mindfulness OR engagement OR sleep OR cognitive clarity OR cognitive function OR body appreciation OR body functionality OR body attunement OR affect OR emotion OR emotions OR enjoyment OR depression OR anxiety OR quality of life OR self-esteem OR self-worth OR body image OR self-efficacy OR attitudes OR social norms OR control OR action plans OR coping OR stress OR decisional balance OR self-schemata OR personality OR knowledge OR health OR barriers OR benefits OR beliefs OR stages of change OR processes of change OR skills OR diet OR smoking OR alcohol OR music OR meditation OR relaxation OR social OR flow OR runners high OR dietary habits OR mood OR psychological health)

Two researchers independently identified potentially eligible studies based on title, abstract, and references, according to the prespecified inclusion/exclusion criteria. The same two researchers independently reviewed the full text of the potentially relevant studies. All discrepancies were resolved by consensus. A third researcher resolved any remaining disagreements.

### Data Extraction

A data extraction form was developed, informed by the PRISMA statement for reporting systematic reviews (Liberati et al., 2009). Data extraction included information about (i) study details (authors, year, country of publication), (ii) participants (age, gender, attrition, and blinding), (iii) study design and setting, (iv) correlates of interest (motivation, reasons, intention, regulations, motives, goals, and gains, as well as vitality, happiness, quality of life, well-being, mood, enjoyment, relaxation, flow, mindfulness, meditation, runners high, engagement, sleep, cognitive clarity or function, body appreciation or functionality or attunement, affect, emotion, depression, anxiety, stress, self-esteem, self-worth, body image, self-efficacy, attitudes, social norms, control, action plans, coping, beliefs, stages or processes of change, skills, decisional balance, self-schemata, personality, knowledge, health, barriers, benefits, diet, smoking, alcohol, music, social, dietary habits, psychological health), (v) intervention length and characteristics, (vi) psychosocial instruments, and (vii) results.

Two researchers independently retrieved the data, and all discrepancies were resolved by consensus. A third researcher resolved any remaining disagreements.

### Assessment of the Risk of Bias in Individual Studies

Two researchers independently assessed the methodological quality and risk of bias of included studies using the National Heart Lung and Blood Institute, National Institute for Health (NHLBINIH) Quality Assessment Tool for Observational Cohort and Cross-Sectional Studies (Thomas et al., 2004). Additional items from the Effective Public Health Practice Project (EPHPP) Quality Assessment Tool for Quantitative Studies (Armijo-Olivo et al., 2012) were added, in order to analyze study aspects exclusively related to interventions. The final tool comprised 20 items, addressing seven key domains: study design; blinding; representativeness (selection bias and withdrawals/dropouts); confounders; data collection; data analysis; and reporting. Each item was classified as present or absent. A global rating of Good (low risk of bias), Fair, or Poor (high risk of bias) methodological quality was determined based on the present or absence of each item (see Supplementary Table 2). Two researchers independently rated each item and overall quality. Discrepancies were resolved by consensus. A third researcher resolved any remaining disagreements.

### Data Synthesis

Participants' sociodemographic characteristics, as well as the psychological and behavioral correlates of running behavior/participation, were qualitatively synthetized and presented in tabular form (Supplementary Table 1). Correlates were divided in antecedents and outcomes, depending on the purpose of the studies and its theoretical frame.

## Results

### Study Selection

**Figure 1:**
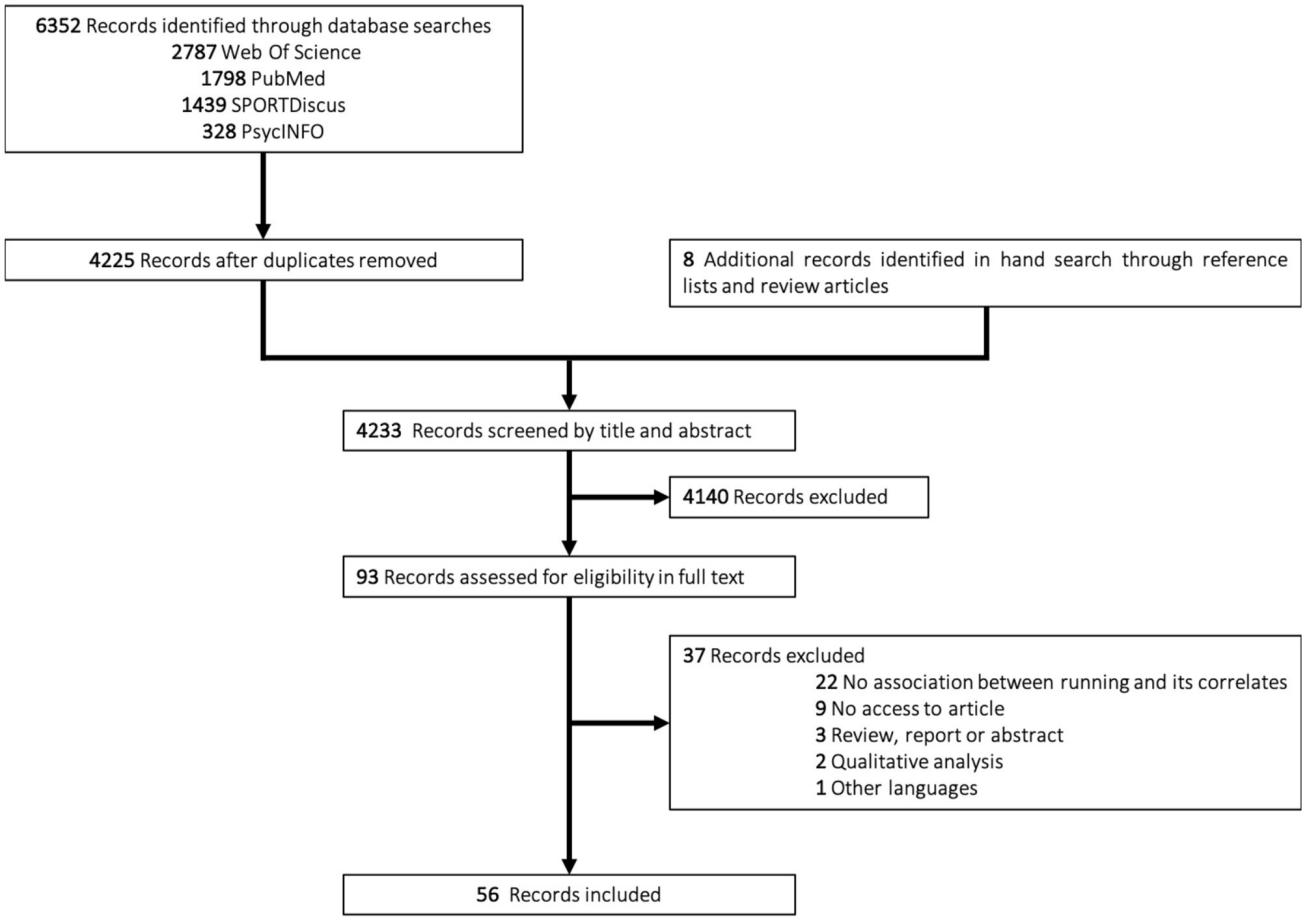
Flowchart of studies.

The literature search yielded 4,225 potentially relevant studies (after duplicates removal). After titles and abstract screening, 4,140 studies were excluded. Common reasons for exclusion were the study design (qualitative study, commentary, or review), not meeting subjects inclusion criteria, and the presence of outcomes related to the preparation for competitive sports (performance-oriented). The full text of the remaining 93 eligible studies was retrieved and reviewed, which, after adding 8 additional records identified through hand search, resulted in the inclusion of 56 articles (see Figure 1).

### Description of Included Studies

There were 27 cross-sectional studies, 12 longitudinal studies, and 19 trials (eight non-controlled trials, five controlled trials, and six randomized controlled trials). Table 2 shows the characteristics of the included studies. In total, 37,501 healthy participants took part in all the studies reviewed, with a range of ages of 1977 years old, and 43% were women. Most studies used samples of regular runners from running groups, communities, or organized events.

**Table 2:** Characteristics of the included studies.

**Study design (*k* = 58)**	**Number of studies**	**Antecedents (*k* = 32)**	**Number of studies**	**Outcomes (*k* = 29)**	**Number of studies**
CS	27	Motives	19	Mood	10
LG	12	Intention	4	Well-being	10
NCT	8	Attitude toward run	3	Depression	6
NRCT	5	Perceived control	3	Anxiety	5
RCT	6	Self-efficacy	3	Cognitive function	4
Sample size		Social support	3	Affect	4
<100	22	Behavior regulation	3	Self-efficacy	3
100199	12	Mood	2	Vitality	3
200299	6	Anxiety	2	Flow	3
>299	18	Depression	2	Stress	2
Participants (*k* = 57)		Subjective norms	2	Perceived health	2
Gender		Self-motivation	1	Life satisfaction	2
Women only	4	Flow	1	Positive orientation	1
Men only	6	Experience	1	Self-esteem	1
Both genders	47	Involvement	1	Enjoyment	1
Age		Shame	1	Fatigue	1
1944	36	Pride	1	Emotion regulation	1
4566	8	Action planning	1		
Broad range	11	Health	1		
QA (*k* = 58)		Process of change	1		
Poor	31				
Fair	20				
Good	7				

*RCT, randomized controlled study; CCT, controlled clinical trial; NCCT, noncontrolled clinical trial; LG, longitudinal study; CS, cross sectional study; QA, quality assessment*.

### Quality of the Studies and Risk of Bias

Thirty-one studies showed poor quality, while 20 had fair and 7 good quality. Generally, the research objective was clear (*k* = 56, 96.6%), subjects were recruited from the same or similar populations (*k* = 52, 89.7%), exposure and outcome measures were clearly defined, valid, reliable, and implemented consistently across all study participants (*k* = 52, 89.7%), and the study population clearly specified and defined (*k* = 37, 78.7%). On the other hand, only two studies presented sample size and power calculations (3.4%), only five had a participation rate of eligible persons of at least 50% at baseline (8.6%), and three had the outcome assessors blinded to the exposure status of participants (5.2%) (Armijo-Olivo et al., 2012) (Supplementary Table 2).

### Synthesis of Results

This systematic review analyzed the published literature describing the psychological and behavioral correlates of recreational running in adults. Overall characteristics of the included studies are presented in Table 2, and in Table 3 the trend of the association with each correlate can be found. A full description and results of individual studies can be found in Supplementary Table 2. In cross-sectional studies, we established *a priori* which correlates were interpreted as antecedents and which were outcomes largely based on information from the study design and methods but also from popular theories of motivation and behavior change, such as the Theory of Planned Behavior (Ajzen and Driver, 1991) or Self-Determination Theory (Ryan and Deci, 2000). Generally, measures of mood and psychological health (depression and anxiety) were treated as consequences of running although they can also play a role in adoption (and that was the case in some longitudinal studies). Twenty-eight studies reported antecedents of running behavior, and 25 studies reported effects or outcomes. Four studies simultaneously examined predictors and outcomes of running.

**Table 3:** Association between the identified correlates and recreational running.

**Antecedents (*k* = 32)**	**Number of studies**	**No association**	**Significant association**		**Outcomes (*k* = 29)**	**Number of studies**	**No association**	**Significant association**	
			**Positive**	**Negative**				**Positive**	**Negative**
Motives	19	13	6		Mood	10	2	8	
Intention	4	2	2		Well-being	10	1	9	
Perceived control	3	1	2		Depression	6	1		5
Attitude toward run	3	1	2		Anxiety	5	2		3
Self-efficacy	3		3		Cognitive function	4	1	3	
Social support	3	2	1		Affect	4	2	2	
Behavior regulation	3	1	2		Self-efficacy	3	2	1	
Mood	2	1	1		Vitality	3	1	2	
Anxiety	2	1		1	Flow	3	2	1	
Depression	2	1		1	Stress	2			2
Subjective norms	2		2		Perceived health	2		2	
Self-motivation	1		1		Life satisfaction	2		2	
Flow	1		1		Positive orientation	1		1	
Experience	1	1			Self-esteem	1	1		
Involvement	1		1		Enjoyment	1		1	
Shame	1	1			Fatigue	1			1
Pride	1		1		Emotion regulation	1	1		
Action planning	1		1						
Health	1		1						
Process of change	1	1							

### Antecedents of Recreational Running

Motives were frequently reported as antecedents of running behavior (*k* = 19). Studies described motive prevalence among different samples of runners, while others studied motives according to running experience, training, gender, and age. Studies of other predictors, such as intention (*k* = 4), perceived control (*k* = 3), attitude toward running (*k* = 3), self-efficacy (*k* = 3), social support (*k* = 3), behavior regulation (*k* = 3), subjective norms (*k* = 2), mood (*k* = 2), measures of trace anxiety and depression, or state anxiety at the beginning of the program (*k* = 2) were also included. Regarding self-motivation, experience, involvement, action planning, process of change, perceived health, flow during the race, shame, and pride, only one study was found for each variable.

Nineteen cross-sectional studies described or compared motives between groups of runners. Among the highest-ranked motives reported were physical health (Krouse et al., 2011), psychological motives (Tjelta et al., 2017), health orientation (Malchrowicz-Moko et al., 2020), and personal achievement (Doppelmayr and Molkenthin, 2004). Less frequent or lower-ranked motives were competition (Leedy, 2000), social motives, such as affiliation or social comparison (Malchrowicz-Moko et al., 2018), and also having fun (Tjelta et al., 2017).

Motives such as competition and health (Ogles and Masters, 2000) or personal goal achievement (Pereira et al., 2021) were associated with weekly training distance, while enjoyment anticipated the adoption of regular running (Titze et al., 2005). Although one study found an interaction between high enjoyment and high family support in the prediction of running behavior (Titze et al., 2005), another found no significant main or interaction effects of social condition (Carnes et al., 2016). One study found that effort was greater for participants who usually reported experiencing more pride than others (Gilchrist et al., 2017).

Studies comparing motives between different groups found that age (Ogles and Masters, 2000), gender (Tjelta et al., 2017), experience in running (Masters and Ogles, 1995), training profile (Ogles et al., 1995), and type of event (Doppelmayr and Molkenthin, 2004) were associated with different motives. Younger runners were more motivated by personal goal achievement and competition (Pereira et al., 2021). In opposition, older runners were more motivated by health orientation, weight concern, life meaning, and affiliation with other runners (Ogles and Masters, 2000). Age was positively associated with health orientation and affiliation and negatively correlated with weight concern, personal goal achievement, competition, recognition, psychological coping, life meaning, and self-esteem (Wakiewicz et al., 2019a). Other authors found that older runners were also more frequently motivated by the exercise itself and experiencing nature, and less by challenge (Tjelta et al., 2017). Nonetheless, older runners who reported competition as an important motive were more likely to have participated in more marathons and trained greater distances per week (Ogles and Masters, 2000).

Regarding gender differences, studies observed that male runners scored higher on competition and challenge (Tjelta et al., 2017) or achievement motives (Whitehead et al., 2020). In agreement, Popov et al. (2019) observed that women scored higher on both mental health improvement and physical health and condition, while men scored significantly higher on the competitive factor. On the opposite, data from another study suggests that women were more likely to endorse psychological coping, self-esteem, and personal goal achievement motives for running (Nikolaidis et al., 2019). Data from other samples showed that compared to men, women scored higher on weight concern, affiliation, psychological coping, life meaning, and self-esteem (Ogles et al., 1995; Wakiewicz et al., 2019a).

A study comparing motives across different levels of running experience suggests that most experienced veterans and runners with mid-level experience scored higher on both competitive and health motives, whereas first-time runners were not characterized by either function (Masters and Ogles, 1995). Others recorded differences in intrinsic motives accordingly with runners' ability. Runners with high and medium ability were most induced by altruism, while runners with low ability were motivated by health (Bell and Stephenson, 2014). A recent study with Polish runners found that running experience was negatively associated with personal goal achievement and self-esteem (Wakiewicz et al., 2019a), while other found no differences in motives according to running experience (Malchrowicz-Moko et al., 2020).

Concerning the amount of training and commitment to running, data suggest that weekly distance was associated with personal goal achievement (Pereira et al., 2021). Similarly, runners registered for a marathon event, running more than 45 miles and intending to continue after the race, were more likely to endorse competition, personal goal achievement, and recognition, as motives for continued training; in turn, less serious runners (registered for a 5K race, not having participated in a marathon, training <15 miles per week and intending to continue after the race) endorsed more of a general health orientation (Masters and Ogles, 1995).

Two studies compared motives for participation of a sample of adventure ultramarathon, ultramarathon, and marathon runners. Results revealed significant differences between the three groups of runners indicating less importance of the reason competition, but higher importance of the motives nature and life meaning for adventure ultramarathon participants compared to marathon runners (Doppelmayr and Molkenthin, 2004). Ultramarathoners showed higher scores in affiliation and life meaning and lower in the areas of weight concern, personal goal achievement, and self-esteem than runners covering shorter distances (Wakiewicz et al., 2019b). On the other hand, 5K runners showed highest scores on self-esteem, physical fitness, and achievement motives (Whitehead et al., 2020).

Ogles and Masters (2003) have found a motivational-based (MOMS) five-cluster solution in 1,519 runners participating in one of the midwestern marathons: Running Enthusiast (RE), Lifestyle Managers (LM), Personal Goal Achievers (PGA), Personal Accomplishers (PAc), and Competitive Achievers (CA). Differences between clusters were significant: CA ran more days per week than LM and PGA; LM trained fewer miles than all the other groups; and RE had completed more marathons than LM, PGA, and PAc.

### Other Antecedents

Concerning intention as an individual's plan to participate in a single behavior, engage in a behavioral category, or achieve a goal, studies suggest that it was positively associated (Bell and Stephenson, 2014) or predicted future running participation (Luszczynska et al., 2007). Others reported that both cognitive (important, relevant, valuable, means a lot, and needed) and affective (interesting, appealing, fascinating, exciting, and involving) elements of the personal involvement inventory were predictors of participation among ultramarathon athletes (Mueller, 2012).

According to one study, behavior at baseline and recovery self-efficacy predicted future participation (Luszczynska et al., 2007). Another study showed correlation between baseline self-efficacy with running and between fluctuation in self-efficacy and fluctuation in running (Scholz et al., 2008). Regarding gains (outcomes runners have already experienced), flow felt in the race was positively correlated with the future running motivation (Schler and Brunner, 2009), and vigor (mood scale) showed correlation with future running behavior (Suter and Marti, 1992).

Two earlier studies with the same sample showed that beliefs, attitudes, norms, and behavior control contributed to behavior prediction (Ajzen and Driver, 1991, 1992). Other studies found some associations between autonomous forms of motivation and both event participation and training compliance. Individuals high in autonomous behavior regulations reported significantly higher levels of participation in both marathons and half marathons (Aicher et al., 2017), and runners with higher self-motivation scores complied better with the exercise regimen (Welsh et al., 1991). Later, two other studies showed that ability, defined as 5K race personal record in the past 2 years standardized by age and gender, was positively associated with participation (Bell and Stephenson, 2014), and there were also correlations between the linear trend action planning, and action control, which are self-regulation skills, with the linear trend in running (Scholz et al., 2008). In the Scholz et al. (2008), a positive correlation emerged between baseline coping planning and linear trend in running over time.

Finally, three studies describing runners behaviors found that 85.2% of runners set goals for their chosen events, 80.1% trained alone and with others (Krouse et al., 2011), more than 90% of runners systematically prepared themselves for the competition (Piot, 2015), 73% of the participants prefer to run alone, 69% do other physical activities besides running, 69% use technology during running sessions, and 68% report running in a natural setting (Pereira et al., 2021).

### Outcomes of Recreational Running

Studies examining outcomes of recreational running assessed psychological outcomes such as mood (*k* = 10), well-being (*k* = 10), affect (*k* = 4), cognitive function (*k* = 4), self-efficacy (*k* = 3), vitality (*k* = 3), flow (*k* = 3), perceived health (*k* = 2), and life satisfaction (*k* = 2). Likewise, some studies reported reductions in depression (*k* = 6), anxiety (*k* = 5), and stress (*k* = 2). Concerning positive orientation (self-esteem, satisfaction with life, and optimism), self-esteem, enjoyment, physical fatigue, and emotion regulation (deficits), only one study was found for each variable.

Studies aiming to understand the effect of running on mood suggest a main effect for running time, immediately after a short run trial (Berger and Owen, 1998; Berger et al., 1998, 2016; Anderson and Brice, 2011). A previous study compared prepost changes in mood and found that the running group exhibited significant changes in total mood disturbance, tension, and confusion immediately after the running session (75 min and more than two miles running) (McGowan and Pierce, 1991). Mood benefits after a marathon race included decreases in depression, anger, confusion, tension, and fatigue and increases in vigor (Hassmn and Blomstrand, 1991).

No significant acute (immediately after, 20 and 40 min post-training) or medium (6 and 9 weeks) term effects of running were observed in one study (Walter et al., 2013). Another RCT found no differences between the acute effect of a 30-min run and equivalent time doing stretching (Bernstein and McNally, 2017). One study found long-term (17 years) improvements in mood states of a small group of runners (Morgan and Costill, 1996).

Well-being was suggested as a positive psychological outcome/correlate of running in a cross-sectional study (Galper et al., 2006). Studies reported acute positive effects of running on revitalization, tranquility, positive engagement, physical exhaustion (Szabo and Abraham, 2013), and positive orientation (Gorczyca et al., 2016). Well-being was cross-sectionally related with motives for running (Popov et al., 2019) and also longitudinally associated with the amount of running (distance and frequency) (Nezlek et al., 2018). However, data suggest that the effect of running on well-being diminishes over time (Bonham et al., 2018).

### Other Outcomes

Contradictory results regarding the flow/worry ratio were found in two randomized controlled trials by the same author (Elbe et al., 2010). While female runners experienced significantly more flow than football players, no differences were observed in males. Worry was higher in male runners than in male football players, but no differences were observed in female. Regarding depression, an inverse association with running has been cross-sectionally observed for both men and women (Galper et al., 2006; Roeh et al., 2020) but also longitudinally: marathon runners showed lower Beck Depression Inventory scores when compared to controls (Winker et al., 2010). The effect of running on anxiety has been addressed in some studies (Larumbe-Zabala et al., 2019). An RCT showed a negative association between state anxiety at program end and running frequency (Welsh et al., 1991). A long-term longitudinal study observed that anxiety decreased significantly across the 23-year period in one sample, while it increased in another. In addition, the neuroticism score for the combined sample decreased significantly (Morgan and Costill, 1996). Others found that both comedy videos and running exercise resulted in reductions of state anxiety (Szabo, 2003). One longitudinal study found that the probability of mental stress (tense, nervous, impatient, anxious, sleepless) was lowest for joggers, when compared with low, moderate, and high physical activity levels (Schnohr et al., 2005).

## Discussion

This review sought to systematically synthesize the published literature describing the psychological and behavioral correlates of recreational running in adults. Because of the limited number of studies reporting each correlate, it was not possible to meta-analyze the data. However, the identification of antecedents most strongly associated with recreational running, such as intrinsic motives, highlights potential candidates to target in future real-world interventions in this domain. Likewise, the identification of most common psychological benefits of running, for instance mood, can strengthen its perceived value and the likelihood of its adoption.

### Antecedents of Recreational Running

The findings from this review show that a typical runner sets goals for specific running events, systematically prepares for competing, and runs for 3050 km/week in average. Studies reporting motives of runners suggest that the highest-ranked or more prevalent motives were physical health, psychological motives, and personal achievement. It can be argued that, due to the item's narrative, intrinsic and extrinsic motives can coexist in the same dimension; nevertheless, health orientation, personal goal achievement, self-esteem, life coping, and life meaning are predominantly intrinsic motives (Gunnell et al., 2014) and were often present among those who sustain their running behavior. Additionally, participants in some of the studies were long-distance runners, using running as a cathartic behavior, often used as a coping mechanism (Nemec, 2016). They are moved by more intrinsic reasons, such as personal achievement and general physical and psychological health. The study which compared motives across adventure ultramarathon, ultramarathon, and marathon runners hints for a lesser importance of competition but higher importance of the contact with nature and life meaning for adventure ultramarathon participants compared to marathon runners (Doppelmayr and Molkenthin, 2004). It could be explained by the unique characteristics of these highly demanding ultramarathons, in which finishing is the main goal. Similar findings emerged from the study comparing obligatory runners, moved by competition, goal achievement, and recognition, with recreational runners, motivated mainly by health purposes (Ogles et al., 1995). Extrinsic motives, like competition or social motives, such as social comparison were indeed less frequent or lower ranked. Since results come from samples of regular runners, which are in behavior maintenance, these findings are in line with the Self-determination Theory assumptions (Ryan and Deci, 2019) and quite similar to those of previous studies of physical activity behavior correlates (Teixeira et al., 2012; Sheeran et al., 2020).

Although both intrinsic motives and autonomous behavior regulations were predictors of higher levels of running participation (Aicher et al., 2017) and perseverance (Qiu et al., 2020), one study (Gilchrist et al., 2017) found that pride, which is a manifestation of introjected behavior regulation (Ryan and Deci, 2000), can motivate people to put forth immediate (5 weeks) and greater effort and persistence toward long-term goals despite short-term costs.

Results suggest that age influences the main reasons why people run (Ogles and Masters, 2000; Tjelta et al., 2017). Generally, older participants are more autonomously motivated than their younger colleagues. Similar results were registered in the comparison of different levels of running experience. Veterans and mid-level experience runners were mostly driven by health, personal goal achievement, and self-esteem. Results match those found in CrossFit participants (Box et al., 2019), in which older participants scored higher on health-related motives, while younger participants scored higher on social motives relative to their counterparts. Others (Molanorouzi et al., 2015) suggest that young adults are also motivated by affiliation, mastery, and enjoyment whereas middle-aged adults considered psychological condition and others' expectations more important motives for participating in PA, than young adults.

Data from two studies indicates gender differences in the motives for running (Ogles et al., 1995; Tjelta et al., 2017). Men tend to run more for competition and challenge, and women for weight concern, affiliation, psychological coping, life meaning, and self-esteem. Similar results were found in a cross-sectional survey about motives for PA, indicating that females reported higher motivation for appearance and physical condition than males, whereas males were more motivated by competition/ego and mastery than females (Molanorouzi et al., 2015).

It was suggested that gains may play a role in running maintenance, regardless of the nature of the expected benefits. Flow experienced in the race and vigor showed correlation with future running behavior. This supports the assumption that gains can function as a reward of the running activity, acting as moderators of the effects of motives, which leads to the desire to perform the activity again (Ingledew et al., 2014).

Ability, beliefs, attitudes, norms, perceived behavior control, and intention positively predicted running participation (Ajzen and Driver, 1991, 1992). This result agrees with the central idea of the Theory of Planned Behavior (Ajzen and Driver, 1991) that once an individual forms the intention to perform a behavior, he or she is extremely likely to actually behave in that manner and that it predicts how hard people are willing to try, and how much of an effort they are planning to exert in order to perform the behavior. However, many fail to translate their physical activity intentions into behavior. This intentionbehavior gap can be explained by (a) explicit trait self-control, (b) implicit executive functions, and (c) their interactions (Pfeffer and Strobach, 2017).

Previous behavior and self-efficacy predicted future participation (Luszczynska et al., 2007; Scholz et al., 2008). These conclusions agree with the principles of the Socio-cognitive Theory (Bandura, 1982). Conversely, the difference in the strength of effects of intention and self-efficacy on behavior may depend on the particular type of self-efficacy. Among individuals who experienced lapses or who decline in their performance, recovery self-efficacy may be a stronger predictor of physical activity than just beliefs about the ability to maintain behavior, because these beliefs themselves are measured in a way more proximal to actual behavior.

The role of action planning and action control for predicting running behavior was also identified (Scholz et al., 2008), suggesting that self-regulation skills may play an important role in running maintenance (Sniehotta et al., 2006; Carraro and Gaudreau, 2013; Reyes Fernandez et al., 2015). Both cognitive (need or importance) and affective (sign value or pleasure) elements of involvement showed to be predictors of participation among ultramarathon athletes. Runners are attracted to the sport through emotion (affective), then build self-perceived skill through facts and problem-solving (cognitive) (Mueller, 2012).

### Outcomes of Recreational Running

There is considerable research on the relationship between exercise and its positive psychological outcomes. Running psychological benefits are interrelated, so differentiating one from another may not be possible. Among several possible causes, self-efficacy, thermogenic effect of exercise, hormonal response, and unclear neurobiological mechanisms are the strongest essential means (Szabo, 2013; Szabo and Abraham, 2013).

Results suggest a positive effect of running on mood after a single exposure. Methods (instruments, samples, and time frame) from the included studies on mood were quite different, but mood benefits included decreases in depression, anger, confusion, tension, and fatigue, as well as increase in vigor (Rendi et al., 2008; Berger et al., 2016). These results agree with previous reviews about the mood-enhancement effect of exercise (Basso and Suzuki, 2017; Chan et al., 2019) and may be partially explained by some neurosteroid blood level changes (Sonnenblick et al., 2018).

Well-being is a general positive psychological outcome associated with running. There is some evidence of a doseresponse relationship. It is clearer regarding frequency than duration, maybe because of the influence of fatigue and physical pain related to long running bouts. It was studied as perceived health, psychological health, revitalization, tranquility, positive engagement, subjective feeling, arousal and positive orientation, and less physical exhaustion and positive orientation (Gorczyca et al., 2016; Bonham et al., 2018). Such findings are coherent with previous evidence that exercise may benefit for people of any age, improving psychological well-being and quality of life (Mandolesi et al., 2018), and can be partially justified by the effect of exercising in a natural environment (Lahart et al., 2019).

In agreement with the flowexercise relationship found in the literature (Jackman et al., 2019), results from one intervention regarding experiences of flow were unsatisfying (Elbe et al., 2010). Results indicate that all groups experience rather high levels of flow regardless of the kind of sport (running or any other).

The registered reduction in depression, anxiety, stress, and life dissatisfaction were to some extent in harmony with the improvement of positive psychological outcomes and with previous systematic reviews of the effect of exercise on anxiety, depression, and quality of life (Ensari et al., 2015; Morris et al., 2018).

Though results suggest that running is associated with improved cognitive function, evidence is still scarce (Batmyagmar et al., 2019). Immediately upon completing the marathon, runners showed impairment in the explicit memory task, but enhancement in the implicit memory task (Eich and Metcalfe, 2009). Likewise, it seems that nonverbal fluency, attention, and neuromotor performance may be increased in runners or as a result of a running intervention, mediated by increased neurotrophic factor release (Harada et al., 2004). These results match those from two recent meta-analyses (Northey et al., 2018; Falck et al., 2019), suggesting that exercise improves cognitive function in both adults and older adults, reiterating the notion that exercise is a panacea for aging well.

These results generally agree with previous reviews of physical activity and exercise correlates. According to our findings, intrinsic motives, goal-setting, and other self-regulation skills seem to be the key antecedents of regular running. Hence, interventions that nurture intrinsic motives and behavior self-regulation skills may increase the likelihood of sustainable adoption of running. On the other hand, improvements in mood and well-being and reductions in depression and anxiety appear to be the main outcomes. For many other variables, studies remain very scarce preventing us from withdrawing firm conclusions regarding their role in recreational running.

This systematic review provides the first comprehensive approach to identify psychological and behavioral correlates of recreational running across populations. Like in other systematic reviews, the variety of studies available (variables, study designs, measurement methods, populations represented, and so forth) is a substantial limitation. In this review, the heterogeneity of research designs, assessment instruments, correlates, populations, and intervention (running) characteristics hindered the process but simultaneously enriched its results.

### Strengths and Limitations

Despite the findings of the review, a number of limitations must be considered. The search strategy was limited to English-language publications, and thus, there is a possibility of a language bias in the systematic review. Unpublished studies and evidence from gray literature were not included, increasing the chance of an incomplete picture of all the studies in this field. Also, due to the heterogeneity of research designs, instruments, correlates, populations, and intervention, it was not possible to conduct a meta-analysis to determine the strength of the correlation between all the correlates and the running behavior itself. Few studies were found for some of the correlates, hindering solid conclusions or interpretations. Finally, many of the included studies had a cross-sectional design, hampering the possibility of causality inference between variables. Future avenues of research could explore further which psychological mechanisms better explain recreational running sustainability and manipulate them but also clarify by which mechanisms running produces its positive outcomes. The popularity of running self-monitoring tools and the possibility to access application programming interface data represent an opportunity to study the association between behavior (measured in multiple data points) and its phycological correlates. To allow the establishment of causality association, longitudinal research designs are recommended. This information can contribute to inform and guide interventions focused on helping people sustain regular physical activity, through an activity they have chosen and appear to enjoy.

## Data Availability Statement

The original contributions presented in the study are included in the article/Supplementary Material, further inquiries can be directed to the corresponding author.

## Author Contributions

HP contributed in the design, search, data extraction, quality assessment, and manuscript. JE and MM contributed for the search, data extraction, quality assessment, and manuscript. AP, IS, EC, and PT contributed in the design, manuscript, and supervision of the overall process. All authors contributed to the article and approved the submitted version.

## Conflict of Interest

The authors declare that the research was conducted in the absence of any commercial or financial relationships that could be construed as a potential conflict of interest.
