# Supplementary material for: Systematic Review of Psychological and Behavioral Correlates of Recreational Running
Source: Front Psychol. 2021 May 7;12:624783. doi: 10.3389/fpsyg.2021.624783 (PMC8139406; doi:10.3389/fpsyg.2021.624783)
Supplement: Supplementary file 1 [file Table_1.docx]

Systematic review of psychological and behavioral correlates of recreational running

**Supplementary table S1.** Description of reviewed studies

| 1. Antecedents of running behavior/ participation | | | | | | | |
| --- | --- | --- | --- | --- | --- | --- | --- |
| Study details | Design | Participants | | Psychosocial instruments | Correlates | Results | QA |
|  |  | Size (%F) | Features |  |  |  |  |
| Aicher et al., 2017, USA | CS | 439 (74.0%) | African American runners (mean 29.4 yrs.) | Revised sport motivation scale (SMS-II) | Behavior regulations | Individuals high in autonomous motivation reported significantly higher levels of participation in both marathons and half marathons. | Fair |
| Bell & Stephenson, 2014, USA | CS | 521 (59.0%) | Completed a 5K race, belonged to running club, or subscribed to a running website (mean 43.0 yrs.) | Runner-ability score, Motives of marathoners scale (MOMS), and Modified Attitude Scale | Motives, Attitude, and Intention | Runners with high ability are most influenced by altruism and competition; Runners with medium ability are most influenced by altruism and social; Runners with low ability are most influenced by health and social. Intention and ability positively predicted participation. | Fair |
| Doppelmayr & Molkenthin, 2004, Austria | CS | 149 (0%) | Adventure ultramarathon, ultramarathon and marathon runners | Motivations of Marathoners Scales (MOMS) | Motives | Differences between the three groups of runners indicating less importance of the reason competition but higher importance of the motives nature and life meaning for adventure ultramarathon participants compared to marathon runners. | Poor |
| Krouse et al. 2011, USA | CS | 344 (100%) | Women ultrarunners 18 years and older (mean 40 yrs.) | Motivation on marathon running scale (MOMS), Perception of success questionnaire (PSQ) and 12 questions designed to distinguish between a task or ego orientation | Motives, Goals | General health orientation and psychological coping were the two strongest motivational factors. Participants were higher in task-orientation (e.g., finishing the race or accomplishing various goals) than ego- orientation (e.g., placing in the top 3 overall or beating an opponent). Trained an average of 12.49 hours a week and spent 64% of their time training alone. Information came from their own experience, blogs, websites. 80% of the participants did not use a coach because of cost and a perceived lack of necessity. Women were task-oriented, internally motivated, health and financially conscious individuals. | Fair |
| Larumbe-Zabala et al., 2019, USA | CS | 140 (12.9%) | Participants in marathon race (mean 39.7 yrs.) | PODIUM questionnaire | Self-confidence, Perceived physical fitness, Social support, Anxiety | Males showed higher values of self-confidence and perceived physical fitness. Women reported higher values of social support. |  |
| Leedy et al. 2000, USA | CS | 276 (62.2%) | Participants in a Midwest running event pre-race dinner (mean 38.3 yrs.) | 19 statements about runners’ motivation, 10 statements from the Running Addiction Scale (RAS) and questionnaire on overall disposition for anxiety and depression.. | Motives, Depression, and Anxiety traits | Stress relief was positively correlated with weekly distance; There was a negative association between depression and adherence to training. Strongest motives were health/fitness and challenge. | Poor |
| Malchrowicz-Mósko et al., 2018, Poland | CS | 178 (41.0%) | University students running half-marathon (19 – 50 yrs.) | Self-constructed motives questionnaire | Motives | Most prevalent motives - maintain good physical/ health condition, check myself, develop passion, achieve the goal set, to experience strong emotions, feel the extraordinary mood of the whole party, feel unity and integration with other people, have fun. | Poor |
| Malchrowicz-Mósko et al., 2020, Poland | CS | 493 (29.2%) | Runners participating in the PKO Poznan Marathon (18-70) | Motivation on marathon running scale (MOMS) | Motives | No differences in motives according to running experience. | Poor |
| Masters & Ogles, 1995, USA | CS | 472 (19.9%) | Runners who participated in one of three Midwestern marathons (54.4 yrs.) | Motivation on marathon running scale (MOMS) | Experience, Motives | Veterans scored significantly higher on Marathon Identity than did the mid-level or rookies. Mid-level group Scored highest on Internal Focus followed by the veterans and rookies. | Poor |
| Mueller et al., 2012, USA | CS | 424 (17.4%) | Subscribers to Ultrarunning magazine, and athletes who entered the Umstead 100-mile endurance run (mean 46.7 yrs.) | Zaichkowsky's Personal Involvement Inventory (PII), Laurent and Kapferer's Consumer Involvement Profile (CIP) | Involvement, and Intention | Cognitive and affective elements of the PII scale were not predictive of intention, but were predictors of participation among ultramarathon athletes. The five CIP factors were predictors of intent to participate. | Poor |
| Nikolaidis et al. 2019, Poland | CS | 166 (19.3%) | Runners participating in the PKO Poznan Marathon (mean 43.4 yrs.) | Motivation on marathon running scale (MOMS) | Motives | Women participants scored higher than male on coping, self-esteem, and personal goal achievement these motives. Younger men (<30 yrs.) scored higher than older ones (35-45 yrs.) on competition motive. The slowest women scored higher on competition and personal goal achievement than the faster ones. |  |
| Ogles et al. 1995, USA | CS | 610 (21.0%) | Runners registering to participate in a midwestern marathon, half-marathon or one of three 5k/10k races. (mean 37.5 yrs.) | Motivations of Marathoners Scales (MOMS) | Motives | Obligatory runners were more likely to endorse competition, personal goal achievement, and recognition, as motives for continued training, whereas the recreational runners endorsed more of a general health orientation. Women scored higher on weight concern, affiliation, psychological coping, life meaning, and self-esteem. | Poor |
| Ogles & Masters, 2000, USA | CS | 214 (0%) | Runners pre-registered for one midwestern marathon (between 20 and 28 or > 50 yrs.) | Motivations of Marathoners Scales (MOMS) | Motives | Older runners were more motivated by health orientation, weight concern, life meaning, and affiliation with other runners. Younger runners were more motivated by personal goal achievement. Older runners who reported competition as an important motive were more likely to have participated in more marathons, and those endorsing competitive motives trained greater distances per week. Competition and Health orientation predicted training miles per week. | Poor |
| Ogles & Masters, 2003, USA | CS | 1519 (18.2%) | Runners participating in one of six Midwestern marathons (15-79 yrs.) | Motivations of Marathoners Scales (MOMS) | Motives | MOMS based cluster analysis yielded five cluster solution: Running Enthusiast, Lifestyle Managers, Personal Goal Achievers, Personal Accomplishers, and Competitive Achievers. Motivational differences between clusters were significant. CA ran more days per week than LM and PGA. LM trained fewer miles than all the others groups, and RE had completed more marathons than LM, PGA and PA. | Poor |
| Pereira et al., 2021, Portugal | CS | 1068 (50%) | Telephone survey to a random national representative sample (18-65 yrs.) | Motivations of Marathoners Scales (MOMS); Behavioral Regulation in Exercise Questionnaire (BREQ-3) | Motives and Behavior regulations | General health orientation, self-esteem , and life meaning were the most predominant motives for running. Younger runners tend to run more for personal goal achievement, which was associated with greater weekly distance. | Fair |
| Popov et al., 2019, Serbia | CS | 289 (46.0%) | Participants in at least one marathon recruited online (36.65 yrs.) | Motivations of Marathoners Scales (MOMS) | Motives | Women scored higher on Mental Health Improvement and Physical Health and Condition, while men scored higher on the Competitive Spirit. The younger age group scored higher on Mental Health Improvement, Physical Health and Condition, and Physical Appearance. | Poor |
| Pišot et al., 2015, Slovenia | CS | 260 (38.1%) | Participants in the 1st Istrian marathon (mean 41.4 yrs.) | Motivations of Marathoners Scales (MOMS) | Motives | Most common motives “to compete with myself”; “to improve health and physical fitness” and “to diminish stress”. | Poor |
| Qiu et al., 2020, China | CS | 300 (30.0%) | Participants in the Hangzhou marathon event (>18 yrs.) | Sport Motivation Scale (SMS-6) | Behavior regulations | Amotivation was negatively associated with perseverance, career, ethos, and identity. Identified regulation was positively associated with perseverance, career, ethos, and benefits. Integrated regulation was positively associated with identity, and intrinsic motivation was positively associated with perseverance, personal effort, career, ethos, and benefits. | Fair |
| Tjelta et al., 2017, Norway | CS | 862 (38.4%) | Web based survey distributed to the “Tresjøersløpet” (“The Three Lace Race”) half-marathon (mean 41.8 yrs.) | Questionnaire on reasons to run | Motives | The major reasons for the participants to regularly practice running was importance to their physical and psychological health, it is fun, and for other health issues. Males ran more for competition and challenge. Older runners were more frequently motivated by the exercise itself and experiencing nature, and less by challenge. | Poor |
| Waśkiewicz et al., 2019a, Poland | CS | 1537 (24.7%) | Polish runners through running websites (>18 yrs.) | Motivations of Marathoners Scales (MOMS) | Motives | Age was positively associated with health orientation and affiliation, and negatively correlated with weight concern, personal goal achievement, competition, recognition, psychological coping, life meaning, and self-esteem. Running experience was negatively associated with personal goal achievement and self-esteem. Female marathon finishers exceeded men on the motivational scales for weight concern, affiliation, psychological coping, life meaning, and self-esteem and they scored lower on competitive motivation. | Poor |
| Waśkiewicz et al., 2019b, Poland | CS | 1537 (24.7%) | Polish runners through running websites (>18 yrs.) | Motivations of Marathoners Scales (MOMS) | Motives | Ultra-marathoners had higher scores in affiliation, life meaning and lower in the areas of weight concern, personal goal achievement and self-esteem than runners covering shorter distances. | Poor |
| Whitehead et al., 2020, UK | CS | 1022 (32.2%) | Sent via email to all participants engaging in a national marathon event (13-77 yrs.) | Reduced version of Motivations of Marathoners Scales (MOMS) | Motives | 5K runners showed highest scores on self-esteem, physical fitness and achievement motives. Males scored higher on achievement motives. | Fair |
| Ajzen & Driver, 1991, USA | LG | 146 (70.6%) | Undergraduate university students (mean 20.1 yrs.) | Attitudes toward behaviors, Subjective norms and perceived behavioral control scales | Attitude towards running, Subjective norms, Perceived behavior control | Attitudes, norms and behavior control contribute to behavior prediction. | Poor |
| Ajzen & Driver, 1992, USA | LG | 146 (70.6%) | Undergraduate university students (mean 20.1 yrs.) | Attitudes toward behaviors, Subjective norms and perceived behavioral control scales | Attitude towards running, Subjective norms, and Perceived behavior control | Beliefs, attitudes, norms and behavior control contribute to behavior prediction. | Poor |
| Gilchrist et al., 2017, USA | LG | 158 (76.0%) | Men and women residing in the Greater Toronto Area and training for a marathon/ half-marathon (mean 32.5 yrs.) | Single-item measures of pride and shame | Shame, and Pride | Effort was greater for participants who usually reported experiencing more pride than others. | Poor |
| Luszczynska et al., 2007, UK, Poland, Germany | LG | 139 (20.1%) | Advertised on a noncommercial website for leisure-time running (mean 29.5 yrs.) | Three questions for intention, four for maintenance self-efficacy, and for recovery self-efficacy | Self-efficacy, and Intention | Intention at T1 predicted behavior measured 2 years later; Behavior at T1 predicted behavior at 2 years; Recovery self-efficacy at T1 predicted behavior 2 years later. | Poor |
| Scholz et al., 2008, Switzerland | LG | 30 (86.7%) | Formerly untrained participants in a training program for running a marathon (mean 41.2 yrs.) | Action and coping planning items, Action control items, Frequency and running volume question, Objective performance data from Gutenberg Marathon | Planning, and Self-efficacy | Baseline self-efficacy was positively associated with baseline running and fluctuation in self-efficacy correlated positively with fluctuation in running. There were correlations between the linear trend action planning, and action control and the linear trend in running. A positive correlation emerged between baseline coping planning and linear trend in running over time. | Fair |
| Titze et al., 2005, Austria | LG | 539 (100%) | Registered for the women’s fun run held in Graz, Austria | Questions about individual and social factors, running motives, and environmental factors, 16 reasons to run, process of change, and running regularity. | Motives, Perceived health, Social support, and Process of change | Predictors of adoption of regular running were process of change frequent use, high enjoyment, and interaction between high enjoyment and high family support. Predictors of regression from of regular running were having few motives, bad perceived health, and interaction between low perceived health and low attractiveness. | Fair |
| Carnes et al., 2016, USA | NCCT | 24 (50.0%) | Participants from 2 running clubs (mean 37.0) | “Liking” visual analog scale, Borg RPE scale, and GPS | Peers influence | There were no significant main or interaction effects of social condition. | Poor |
| Schüler & Brunner, 2009, Switzerland | NCCT | 109 (17.4%) | Participants recruited at a marathon exhibition prior to a race (mean 36.3 yrs.) | Flow Short Scale, and three question about future running motivation | Flow | Mean flow experience during the race was positively correlated with the future running motivation. | Fair |
| Suter & Marti, 1992, Switzerland | RCT | 61 (0%) | Volunteers wishing to enter an exercise program | Running diaries, and Mood questionnaire | Mood | Vigor (mood scale) at 4 months showed correlation with running at 8 months, but lack of energy and depressiveness did not. | Good |
| Welsh et al., 1991, USA | RCT | 22 (100%) | Women were recruited from a community to begin a jogging program (mean 35.7 yrs.) | Multidimensional-health locus of control, Jenkins activity survey; Self-motivation inventory | Anxiety, Depression, Locus of control, and Self-motivation | Subjects with higher self-motivation scores complied better with the exercise regimen. | Good |
| 1. Outcomes of running behavior/ participation | | | | | | | |
| Study details | Design | Participants | | Psychosocial instruments | Correlates | Results | QA |
|  |  | Size (%F) | Features |  |  |  |  |
| Eich & Metcalfe, 2009, USA | CS | 261 (34.9%) | Have completed either the NYC or Boston Marathon | Modified Graf & Williams’s Normed Word Pool | Memory | There was an interaction between group (marathon vs. control) and memory task (implicit vs. explicit). Explicit memory task was worse and implicit memory test was better for the marathon group. | Poor |
| Galper et al., 2006, USA | CS | 6555 (19.4%) | Aerobics Center Longitudinal Study population (mean 49.2 yrs.) | Center for epidemiologic studies depression scale (CES-D), and General well-being schedule (GWB) | Emotional wellbeing and depression | Negative association between running and estimated mean CES-D scores for both men and women, and positive association with estimated mean GWB scores in men and women. | Fair |
| Larumbe-Zabala et al., 2019, USA | CS | 140 (12.9%) | Participants in marathon race (mean 39.7 yrs.) | PODIUM questionnaire | Anxiety | Women reported higher values of anxiety. |  |
| Pereira et al., 2021, Portugal | CS | 1068 (50%) | Telephone survey to a random national representative sample (18-65 yrs.) | Dispositional Flow Scale-2 (DFS-2); Subjective Vitality Scale (SVS) | Vitality and Flow | Vitality and flow showed positive association with life meaning, general health orientation, identified and integrated behavior regulation, and intrinsic motivation. | Fair |
| Popov et al., 2019, Serbia | CS | 289 (46.0%) | Participants in at least one marathon recruited online (36.65 yrs.) | Serbian Inventory of Affect (SIAB-PANAS); Short Subjective Well-Being Scale | Wellbeing and Affect | Mental health improvement motives were negatively associated with positive affect and well-being, and positively related to negative affect. Stress Coping, Affiliation and Physical Health and Conditioning were positively associated with Positive Affect. Physical Health and Condition was also a significant predictor of Well-Being. | Poor |
| Ransford & Palisi, 1996, USA | CS | 2980 (62.0%) | Data from The National Survey of Personal Health Practices and Consequences (20-64 yrs.) | Subjective health single item, Psychological well-being 7- item scale | Subjective health and wellbeing | 92% of the males and 88% of females who run often, 71% of males and 90% of women who run sometimes perceived their health and wellbeing as good or excellent. | Poor |
| Roeh et al., 2020, Germany | CS | 106 (25.5%) | Population was part of the ReCaP trial, a longitudinal observational study of marathon runners registered for the Munich Marathon (18-60 yrs.) | Minnesota Multiphasic Personality Inventory 2 (MMPI-2-RF); The self-rating scale Beck Depression Inventory (BDI); and Hamilton Depression Scale (HAMD) | Depression | Marathon runners had lower scores in scales measuring somatic and cognitive complaints, stress, demoralization, hopelessness and distrust. |  |
| Winker et al., 2010, Austria | CS | 114 (10.5%) | Recruited if (a) participated in at least one marathon in the preceding two years, (b) were in continuous training during the recruitment phase and (c) over the age of 60 (mean 66 yrs.) | Mini Mental State Examination (MMSE), Clock Drawing Test, self-rating scales and forms to assess premorbid intelligence levels, subjective memory functions, psychological and physiological well-being, depression. | Cognitive function, wellbeing, psychological health, and depression | Nonverbal Fluency, attention (Symbol Counting Task) was higher in athletes. Marathon runners showed higher wellbeing, and psychological health, and lower Beck Depression Inventory scores when compared to controls. | Poor |
| Batmyagmar et al., 2019, Austria | LG | 99 (9.1%) | Elderly marathon runners (mean 66 yrs.) | German Plus-version of neuropsychological test battery, Short form health survey (SF-36) | Cognitive function and subjective health | Cognitive performance was not better in athletes than in non-athletes. Self-reported health is higher in endurance athletes compared to non-athletes. | Fair |
| Bonham et al., 2018, UK | LG | 38 (47.4%) | Parkrun recreational runners (18 - 50 yrs.) | Feeling Scale, and Felt Arousal Scale | Affect | Mean valence was significantly higher on run days than next days; higher on next days than baseline days; higher after run than before run; and arousal - Arousal was significantly higher on run days than baseline. | Good |
| Gorczyca et al. 2016, Poland | LG | 80 (29.0%) | Runners who completed the first marathon (mean 35.1 yrs.) | General self-efficacy scale, and Positivity scale | Self-efficacy, positive orientation | PO and GSE were higher after completion of a marathon. | Fair |
| Morgan & Costill, 1996, USA | LG | 15 (0%) | (mean 50.5 yrs.) | Depression Adjective Check List (DACL), Eight-Parallel-Anxiety-Battery, Eysenck Personality lnventory (EPI), Parallel-form anxiety battery (IPAT 8), State-trait anxiety inventory (STAI), Body awareness scale (BAS), and Profile of mood states (POMS) | Depression, anxiety, and mood | Anxiety decreased significantly across the 23-year period, Trait anxiety was observed to increase significantly. neuroticism score for the combined sample decreased significantly. The overall mood score did not differ across time. | Poor |
| Nezlek et al., 2018, Poland | LG | 212 (48.1%) | Residents of Poland recruited via the internet and running magazines (mean 32.5 yrs.) | Rosenberg self-esteem scale, Satisfaction with life scale, affect was based on a circumplex model (e.g., Feldman Barrett & Russell, 1998), questions about life stress | Wellbeing, self-esteem, life satisfaction, self-efficacy, and affect | Well-being was positively related to days people ran and how far they ran each week. Satisfaction with one’s progress mediated relationships between well- being and the amount of running. | Good |
| Schnohr et al., 2005, Denmark | LG | 12028 (54.5%) | Random sample drawn from the Copenhagen Population (mean 55.9 yrs.) | Questionnaire about physical activity, smoking, alcohol consumption, two questions about stress and one about life dissatisfaction | Wellbeing, life dissatisfaction and stress | There was a clear trend of higher level of stress and life dissatisfaction in the sedentary group compared with the more active groups. There was a dose–response effect between physical activity and psychosocial well-being. | Fair |
| Berger & Owen, 1998a, USA | NCCT | 71 (54.9%) | College students enrolled in 3 body conditioning courses (mean 21.4 yrs.) | Profile of mood states (POMS) | Mood | Joggers reported mood benefits (tension, depression, anger and confusion). | Poor |
| Berger & Owen, 1998b, USA | NCCT | 68 (58.8%) | College students enrolled in 3 body conditioning courses (mean 22.2 yrs.) | Profile of mood states (POMS) | Mood | Joggers reported mood benefits (tension, depression, vigor and confusion). | Poor |
| Berger et al. 2016, USA | NCCT | 55 (49.1%) | College students from three jogging classes (mean 20.6 yrs.) | Profile of Mood States (POMS), and Physical Activity Enjoyment Scale (PACES) | Mood, and enjoyment | Mood benefits included decreases in Depression, Anger and Confusion, and increases in Vigor. | Poor |
| Hassmén & Blomstrand, 1991, Sweden | NCCT | 106 (0%) | Runners with the intention of finishing the Stockholm marathon race in a 3h to 3h45min (mean 40 yrs.) | Profile of mood states (POMS) | Mood | All groups showed decreases in tension and vigor, as well as an increase in fatigue when their pre- and post-marathon scores were compared. | Fair |
| Rendi et al., 2008, Hungary | NCCT | 80 (5.0%) | Regular users of a large urban fitness center (mean 35 yrs.) | Exercise-Induced Feeling Inventory (EFI) | Vitality, and wellbeing | Both groups had high self-reported level of enjoyment of the exercise sessions and in the psychological improvements from pre- to post-exercise (revitalization, tranquility, positive engagement, physical exhaustion). | Fair |
| Szabo & Ábrahám, 2013, Hungary | NCCT | 50 (26.0%) | Every third runner preparing to start her or his run on a specially designated 5-km long public running path (mean 29.0 yrs.) | Exercise-Induced Feeling Inventory (EFI) | Vitality, and wellbeing | Pre- to post- exercise improvements in revitalization, positive engagement and tranquility, and increases in exhaustion. | Good |
| Anderson & Rice, 2011, UK | CCT | 40 (50.0%) | Opportunity sampling from sports clubs (18-25 yrs.) | Short Profile of Mood States (POMS) | Mood | 10-min bouts of exercise beneficially impacted mood | Poor |
| Berger & Owen, 1998c, USA | CCT | 91 (55.0%) | College students enrolled in 3 body conditioning courses and health science class as control. | Profile of mood states (POMS) | Mood | Joggers (regardless of exercise intensity) reported significant reductions in tension, depression, anger, fatigue and confusion, and increases in vigor. | Fair |
| Harada et al. 2004, Japan | CCT | 14 (43.0%) | Volunteers were solicited from the community of the City of Handa (mean 27 yrs.) | Branching task (BR) | Cognitive function | Neuro-motor performance increased from weeks 0 to 6 in the jogging group, but not in control. From weeks 6 to 12, the rates did not increase in either group. | Poor |
| McGowan et al., 1991, USA | CCT | 25 | Student volunteers | Profile of mood states (POMS) | Mood | The running group exhibited significant reductions in total mood disturbance, tension, and confusion. | Poor |
| Szabo et al., 2003, UK | CCT | 39 (43.6%) | Second year sport science students (20-23 yrs.) | Spielberger State Anxiety Inventory (SSAI); Subjective Exercise Experience Scale (SEES) | State anxiety, positive wellbeing, psychological distress, and physical fatigue | Both exercise session and the humor session showed positive results on state anxiety. | Good |
| Bernstein & McNally, 2017, USA | RCT | 80 (50.0%) | Harvard University Study Pool (mean 22.3 yrs.) | Exercise Addiction Inventory: Short Form (EAI); Affect Intensity Measure (AIM), Emotion Regulation Questionnaires (ERQ), and Ruminative Responses Subscale of the Response Style Questionnaire (RRS); The Depression Anxiety Stress Scales (DASS- 21); Affective circumflex measure to report their affect, Difficulties in Emotion Regulation Scale (DERS); Coping Self-Efficacy Scale (CSE). | Emotional regulation, affect, mood, and self-efficacy, depression and anxiety | Before and after 30 minutes of stretching or jogging, the two groups did not differ in their reports of sadness, happiness, overall positive affect (feeling excited, happy, or content), or overall negative affect (feeling anxious, angry, or sad). | Good |
| Elbe et al. 2010a Denmark | RCT | 41 (100%) | Recruited from advertisements in the local newspapers (mean 37.1 yrs.) | Flow Short-scale (FSS) | Flow | Runners experiencing significantly more flow than football players. No differences were found regarding their experience of worry. | Fair |
| Elbe et al. 2010b, Denmark | RCT | 36 (0%) | Recruited from advertisements in the local newspapers (mean 31.8 yrs.) | Flow Short-scale (FSS) | Flow | Worry is higher in runners than in football players. No significant differences were found regarding the flow total score. | Fair |
| Walter et al., 2013, Germany | RCT | 23 (52.2%) | Apprentices from the Karlsruhe Institute of Technology (mean 19.4 yrs.) | Short-scale for mood states | Mood | All mood dimensions increased immediately after acute endurance exercise but results were not significant | Good |
| Welsh et al., 1991, USA | RCT | 22 (100%) | Women were recruited from a community to begin a jogging program (mean 35.7 yrs.) | State-trait anxiety inventories (STAI Form X), Beck depression inventory, Jenkins activity survey | Anxiety and Depression | There was a correlation between state anxiety scores and exercise frequency at baseline and program end. | Good |

RCT - Randomized controlled study; CCT - Controlled clinical trial; NCCT – Non-controlled clinical trial; LG - Longitudinal study; CS - Cross sectional study
