# Supplementary material for: Systematic Review of Psychological and Behavioral Correlates of Recreational Running
Source: Front Psychol. 2021 May 7;12:624783. doi: 10.3389/fpsyg.2021.624783 (PMC8139406; doi:10.3389/fpsyg.2021.624783)
Supplement: Supplementary file 2 [file Table_2.docx]

Systematic review of psychological and behavioral correlates of recreational running

**Supplementary table S2.** Methodological quality and risk of bias assessment of included studies.

|  | Research question | Study population | Participation rate | Subject recruitment | Inclusion and exclusion criteria | Sample size justification | Exposure measured prior to the outcome | Sufficient timeframe | Different levels of the exposure | Clearly defined, valid, reliable exposure measures | Exposure assessed more than once over time | Clearly defined, valid, reliable outcome measures | Outcome assessors blinded | Attrition 20% or less | Confounding variables measured and adjusted statistically | Study design | Randomized | Method of randomization described | Appropriate method | Representative of the target population | Overall Quality |
| --- | --- | --- | --- | --- | --- | --- | --- | --- | --- | --- | --- | --- | --- | --- | --- | --- | --- | --- | --- | --- | --- |
| Aicher, 2017 | Y | Y | N | Y | Y | N | N | N | N | Y | N | Y | Y | NA | Y | CS | N | NA | NA | Y | FAIR |
| Ajzen, 1991 | Y | Y | Y | Y | Y | N | N | Y | Y | Y | N | Y | N | Y | N | LG | N | NA | NA | N | POOR |
| Ajzen, 1992 | Y | Y | CD | Y | Y | N | N | Y | Y | Y | N | Y | N | Y | N | LG | N | NA | NA | N | POOR |
| Anderson, 2011 | Y | N | CD | Y | Y | N | N | N | N | Y | N | Y | CD | CD | N | BAS | Y | Y | Y | N | POOR |
| Batmyagmar, 2019 | Y | N | CD | CD | CD | N | Y | Y | N | Y | N | Y | CD | Y | N | LG | N | NA | NA | N | FAIR |
| Bell, 2014 | Y | Y | N | Y | Y | N | N | N | NA | Y | N | Y | Y | NA | N | CS | N | NA | NA | Y | FAIR |
| Berger, 1998a | Y | Y | CD | Y | Y | N | N | Y | Y | Y | Y | Y | N | CD | N | BAS | N | NA | NA | N | POOR |
| Berger, 1998b | Y | Y | CD | Y | Y | N | N | Y | Y | Y | Y | Y | N | CD | N | BAS | N | NA | NA | N | POOR |
| Berger, 1998c | Y | Y | CD | Y | Y | N | N | Y | Y | Y | Y | Y | N | CD | Y | CCT | N | NA | NA | N | FAIR |
| Berger, 2016 | Y | Y | CD | Y | Y | N | N | N | Y | Y | N | Y | N | CD | Y | BAS | N | NA | NA | N | POOR |
| Bernstein, 2017 | Y | Y | CD | Y | Y | N | N | N | Y | Y | N | Y | N | CD | Y | RCT | Y | Y | Y | N | GOOD |
| Bonham, 2018 | Y | Y | CD | Y | Y | N | Y | Y | Y | Y | Y | Y | Y | Y | Y | LG | N | NA | NA | N | GOOD |
| Carnes, 2016 | CD | CD | N | Y | N | N | N | N | N | Y | CD | Y | CD | Y | Y | BAS | Y | N | CD | N | POOR |
| Doppelmayr, 2004 | Y | N | CD | Y | CD | N | N | N | N | Y | N | Y | Y | NA | N | CS | NA | NA | NA | N | POOR |
| Eich, 2009 | Y | N | CD | Y | CD | N | N | N | N | Y | N | Y | CD | NA | Y | CS | N | NA | NA | Y | POOR |
| Elbe, 2010a | Y | Y | CD | Y | Y | N | N | Y | Y | Y | Y | Y | CD | CD | Y | RCT | Y | N | NA | N | FAIR |
| Elbe, 2010b | Y | Y | CD | Y | Y | N | N | Y | Y | Y | Y | Y | CD | CD | Y | RCT | Y | N | NA | N | FAIR |
| Galper, 2006 | Y | Y | CD | Y | Y | N | Y | N | Y | Y | N | Y | N | NA | Y | CS | N | NA | NA | Y | FAIR |
| Gilchrist, 2017 | Y | Y | CD | Y | Y | N | N | N | Y | N | N | N | N | CD | Y | LG | N | NA | NA | N | POOR |
| Gorczyca, 2016 | Y | N | CD | Y | Y | N | Y | Y | Y | Y | Y | Y | N | N | Y | LG | N | NA | NA | N | FAIR |
| Harada, 2004 | N | Y | CD | N | Y | N | Y | Y | CD | Y | Y | Y | CD | Y | CD | CCT | N | NA | NA | N | POOR |
| Hassmen, 1991 | Y | Y | CD | Y | Y | N | Y | Y | Y | Y | Y | Y | N | CD | Y | BAS | N | NA | NA | N | FAIR |
| Krouse, 2011 | Y | Y | N | Y | Y | N | N | N | N | Y | N | Y | N | NA | N | CS | N | NA | NA | Y | FAIR |
| Larumbe-Zabala, 2019 | Y | Y | CD | Y | CD | N | N | N | N | Y | N | N | CD | NA | N | CS | N | NA | NA | N | POOR |
| Leedy, 2000 | Y | Y | CD | Y | Y | N | N | N | Y | Y | N | Y | N | CD | Y | CS | N | NA | NA | Y | POOR |
| Luszczynska, 2007 | Y | Y | CD | Y | N | N | Y | Y | Y | Y | Y | Y | CD | N | N | LG | N | NA | NA | N | POOR |
| Malchrowicz-Mósko, 2018 | Y | Y | CD | Y | Y | Y | N | N | NA | N | N | N | N | NA | N | CS | N | NA | NA | Y | POOR |
| Malchrowicz‐Mośko, 2020 | Y | Y | CD | Y | CD | N | N | N | Y | Y | N | Y | CD | NA | N | CS | N | NA | NA | Y | POOR |
| Masters, 1995 | Y | Y | N | Y | Y | N | N | N | NA | NA | N | Y | CD | NA | N | CS | N | N | N | Y | POOR |
| McGowan, 1991 | Y | N | CD | Y | Y | N | N | N | N | Y | N | Y | N | CD | Y | CCT | N | NA | NA | N | POOR |
| Morgan, 1996 | Y | Y | CD | CD | CD | N | N | Y | Y | Y | Y | Y | CD | Y | N | CHS | N | NA | NA | N | POOR |
| Mueller, 2012 | Y | Y | N | Y | Y | N | N | N | Y | Y | N | Y | N | NA | Y | CS | N | NA | NA | N | POOR |
| Nezlek, 2018 | Y | Y | CD | Y | Y | N | Y | Y | Y | Y | Y | Y | N | N | Y | LG | N | NA | NA | Y | GOOD |
| Nikolaidis, 2019 | Y | Y | CD | Y | CD | N | N | N | Y | Y | N | Y | CD | NA | Y | CS | N | NA | NA | N | FAIR |
| Ogles, 1995 | Y | N | N | Y | N | N | N | N | Y | Y | N | Y | N | NA | N | CS | N | N | N | Y | POOR |
| Ogles, 2000 | Y | N | N | Y | N | N | N | N | Y | Y | N | Y | N | NA | Y | CS | N | N | N | Y | POOR |
| Ogles, 2003 | Y | Y | CD | Y | N | N | N | N | Y | Y | N | Y | N | NA | Y | CS | N | N | N | Y | POOR |
| Pereira, 2021 | Y | Y | Y | Y | Y | Y | N | N | Y | Y | N | Y | N | NA | Y | CS | N | NA | NA | Y | FAIR |
| Pišot, 2015 | Y | N | CD | Y | Y | N | N | N | N | Y | N | Y | N | NA | N | CS | N | NA | NA | Y | POOR |
| Popov, 2019 | Y | Y | CD | Y | CD | N | N | N | Y | Y | N | Y | N | NA | Y | CS | N | NA | NA | N | POOR |
| Qiu, 2020 | Y | Y | CD | Y | Y | N | N | N | Y | Y | N | Y | CD | NA | Y | CS | N | NA | NA | Y | FAIR |
| Ransford, 1996 | Y | Y | CD | Y | Y | N | N | N | N | N | N | N | N | NA | Y | CS | N | NA | NA | Y | POOR |
| Rendi, 2008 | Y | N | CD | CD | N | N | Y | Y | Y | Y | Y | Y | N | Y | N | BAS | Y | Y | N | N | FAIR |
| Roeh, 2020 | Y | Y | CD | N | Y | N | N | N | Y | Y | N | Y | N | NA | N | CS | N | NA | NA | N | POOR |
| Schnohr, 2005 | Y | Y | Y | CD | CD | N | Y | Y | Y | Y | Y | CD | CD | CD | N | LG | N | NA | NA | Y | FAIR |
| Scholz, 2008 | Y | Y | Y | Y | N | N | Y | Y | Y | Y | Y | Y | N | Y | N | LG | N | N | N | N | FAIR |
| Schüler, 2009 | Y | Y | CD | Y | CD | N | Y | Y | Y | Y | Y | Y | N | Y | N | BAS | N | N | N | N | FAIR |
| Suter, 1992 | Y | Y | CD | Y | Y | N | Y | N | Y | N | Y | Y | N | Y | Y | RCT | Y | N | NA | N | GOOD |
| Szabo, 2003 | Y | Y | CD | Y | Y | N | N | N | Y | Y | N | Y | N | CD | Y | CCT | Y | Y | Y | N | GOOD |
| Szabo, 2013 | Y | Y | CD | Y | Y | N | Y | Y | Y | Y | Y | Y | N | CD | N | BAS | Y | Y | Y | N | GOOD |
| Titze, 2005 | Y | Y | Y | Y | CD | N | Y | Y | Y | Y | Y | Y | CD | Y | N | LG | N | N | N | Y | FAIR |
| Tjelta, 2017 | Y | Y | N | Y | Y | N | N | N | N | N | N | N | N | NA | N | CS | N | NA | NA | Y | POOR |
| Walter, 2013 | Y | Y | CD | Y | CD | N | Y | Y | Y | Y | Y | Y | N | Y | N | RCT | Y | Y | Y | N | GOOD |
| Waśkiewicz, 2019a | Y | N | CD | Y | Y | N | N | N | Y | Y | N | Y | N | NA | Y | CS | N | NA | NA | Y | POOR |
| Waśkiewicz, 2019b | Y | N | CD | Y | Y | N | N | N | Y | Y | N | Y | N | NA | Y | CS | N | NA | NA | Y | POOR |
| Welsh, 1991 | Y | Y | CD | Y | Y | N | Y | Y | Y | Y | Y | Y | N | Y | Y | RCT | Y | N | NA | N | GOOD |
| Whitehead, 2020 | Y | Y | CD | Y | Y | N | N | N | Y | Y | N | Y | N | NA | Y | CS | N | NA | NA | Y | FAIR |
| Winker, 2010 | Y | Y | CD | Y | CD | N | N | N | Y | Y | N | Y | CD | Y | N | LG | N | N | N | N | POOR |

Y – yes; N – No; CD – Cannot determine; NA – Non applicable
